# Using mating-type loci to improve taxonomy of the *Tuber indicum* complex, and discovery of a new species, *T*. *longispinosum*

**DOI:** 10.1371/journal.pone.0193745

**Published:** 2018-03-28

**Authors:** Akihiko Kinoshita, Kazuhide Nara, Hiromi Sasaki, Bang Feng, Keisuke Obase, Zhu L. Yang, Takashi Yamanaka

**Affiliations:** 1 Kyushu Research Center, Forestry and Forest Products Research Institute, Kurokami, Kumamoto, Kumamoto, Japan; 2 Graduate School of Frontier Sciences, The University of Tokyo, Kashiwanoha, Kashiwa, Chiba, Japan; 3 Mycologist Circle of Japan, Fujisawa, Kanagawa, Japan; 4 Key Laboratory for Plant Diversity and Biogeography of East Asia, Kunming Institute of Botany, Chinese Academy of Sciences, Kunming, Yunnan, China; 5 Department of Mushroom Science and Forest Microbiology, Forestry and Forest Products Research Institute, Matsunosato, Tsukuba, Ibaraki, Japan; University of Florida, UNITED STATES

## Abstract

Black truffles that morphologically resemble *Tuber indicum* have been known to occur in Japan since 1979. Our previous studies showed that there are two phylotypes of these truffles, both of which fell into the *T*. *indicum* complex (hereinafter “*Tuber* sp. 6” and “*Tuber* sp. 7”). However, their taxonomic treatment is still unclear. In this study, we conducted morphological and phylogenetic analyses for a total of 52 specimens from Japan (16 *Tuber* sp. 6 and 13 *Tuber* sp. 7), China (10 *T*. *himalayense* and 8 *T*. *indicum*), and Taiwan (5 *T*. *formosanum*). We compared ascospore ornamentation, size, distribution of asci with average number of spores per ascus, spine size and shape of the Japanese specimens with their allied taxa. For phylogenetic analysis, we sequenced two mating loci (MAT1-1-1 and MAT1-2-1) and three commonly used loci (ITS, β-tubulin, and TEF1-α). Three distinct lineages were recognized by phylogenetic analyses based on the sequences of the two mating-related loci and three independent loci. The *Tuber* sp. 6 sequences clustered with those of *T*. *himalayense* and *T*. *formosanum*, and there was no clear difference in morphology among them. *Tuber* sp. 7 formed a distinct lineage in each phylogram. The specimens tended to have five-spored asci more frequently than other allied species and could be characterized as having ascospore ornamentation with longer spines and narrower spine bases. We therefore described *Tuber* sp. 7 as a new species (*T*. *longispinosum*), and treat *Tuber* sp. 6 and *T*. *formosanum* as synonyms of *T*. *himalayense*.

## Introduction

Truffles (*Tuber* spp.) are ectomycorrhizal ascomycetes that belong to Pezizales. The hypogeous fruitbodies formed by several species are renowned as highly valued edible mushrooms (e.g., *T*. *magnatum* Pico and *T*. *melanosporum* Vittad.). The prized black truffle *T*. *melanosporum* has been cultivated within its indigenous areas (Europe), but also in non-native regions (e.g., North America and New Zealand) [1,2]. As alternatives to the European black truffle, Asian black truffles have been imported into Europe since the early 1990s and sold at local markets [3].

To date, four Asian black truffle species have been accepted: *T*. *indicum* Cooke & Massee, *T*. *himalayense* B.C. Zhang & Minter, *T*. *formosanum* H.T. Hu & Y. Wang, and *T*. *pseudohimalayense* Moreno, Manjón, J. Díez & García-Mont. [4–7]. However, substantial similarities in ascomata and ascospore morphology make species identification uncertain [8–10]. Morphological and phylogenetic analyses showed that *T*. *pseudohimalayense* and *T*. *pseudoexcavatum* are a single species distinct from *T*. *indicum* [5,11]; and *T*. *indicum* was mainly divided into two groups: *T*. *indicum* groups A and B [9,12,13]. However, the taxonomic treatment of the two groups has still remained controversial. Some researchers have proposed that the two groups (A and B) should be assigned into two distinct species, *T*. *indicum* and *T*. *himalayense*, respectively [13,14,15], whereas others have suggested that they are two ecotypes of *T*. *indicum* [9,10].

Our phylogenetic analyses based on internal transcribed spacer (ITS) sequences of nuclear ribosomal DNA showed that Japanese truffles were composed of 20 phylotypes, which for convenience we denoted as *Tuber* sp. 1 to 20 [16]. Among these truffles, black truffles included two phylotypes, both of which belong to the *T*. *indicum* complex. *Tuber* sp. 6 clustered with *T*. *indicum* group B and *T*. *formosanum* with >98% sequence similarities, whereas *Tuber* sp. 7 is sister to *T*. *indicum* group A with <95% ITS similarities [16]. By taking into account phylogenetic concepts of species delimitation [17] and ITS divergence [18,19], *Tuber* sp. 6 is identical to *T*. *indicum* B and *T*. *formosanum*, whereas *Tuber* sp. 7 is a distinct new species. However, additional anatomical descriptive work is needed for the undescribed species.

Recently, Belfiori et al. [20] showed that both *T*. *indicum* groups A and B, and *T*. *melanosporum* are heterothallic [21,22], which indicates that compatible mating types (MAT1-1 and MAT1-2) are necessary for sexual reproduction. They revealed that the differences in the sequence and organization of the MAT idiomorphs (MAT1-1 and MAT1-2) between *T*. *melanosporum* and each of the two *T*. *indicum* groups showed similar divergence levels. MAT genes are indirectly affected in a speciation event, and the apparent divergences may signal the presence of cryptic species in the *T*. *indicum* complex [20]. Moreover, because mating-type genes appear to evolve faster than other regions in the genome, they have been used as tools to delimit species [23–25], even within a species complex [26,27]. Analysis of mating-type genes should be useful for elucidating the complex taxonomy of the *T*. *indicum* complex [20,28].

In the present study, we aimed to resolve the taxonomy of the Japanese black truffles (*Tuber* sp. 6 and *Tuber* sp. 7, [16]) based on molecular and morphological analyses that included specimens of all related Asian species in the *T*. *indicum* complex. We selected a total of 52 specimens that originated from Japan (*Tuber* sp. 6 and *Tuber* sp. 7), China (*T*. *himalayense* and *T*. *indicum*), and Taiwan (*T*. *formosanum*). This is the first study to present MAT phylogenies for the *T*. *indicum* complex and successfully apply these findings to discriminate a new species.

## Materials and methods

### Sample collection

We examined 16 *Tuber* sp. 6 and 13 *Tuber* sp. 7 collections from our previous phylogenetic studies [16] and additional samples. These specimens spanned a wide geographic range in Japan. For Chinese specimens, 8 *T*. *indicum* group A and 10 *T*. *indicum* group B specimens were selected that were previously used for a population study by Feng et al. [15]. Previous studies showed that *T*. *indicum* groups A and B corresponded to *T*. *indicum* and *T*. *himalayense*, respectively [8,14,15]; we therefore followed their taxonomic treatment. For Taiwanese specimens, five dried *T*. *formosanum* specimens, including the holotype (KUN-HKAS62628) and a paratype (KUN-HKAS48268), were examined (Table 1).

**Table 1:** Voucher sample information and GenBank accession numbers of sequence data used in this study.

Taxa	Herbarium voucher; isolate	Locality	GenBank accession no.				
			ITS	β-tublin	MAT1-1-1	MAT1-2-1	TEF1-α
***T*. *longispinosum***	K204	Miyazaki, Japan	AB553412	LC312239		LC312318	LC312276
**(= *Tuber* sp. 7)**	TFM:S17007; K209*	Ehime, Japan	AB553413	LC312240	LC312353	LC312319	LC312277
	TFM:S17010; K225*	Oita, Japan	AB553414	LC312241			LC312278
	TFM:S17005: K395*	Shizuoka, Japan	AB553418			LC312320	LC312279
	TFM:S17008; K401*	Oita, Japan	AB553420	LC312242	LC312354	LC312321	LC312280
	TFM:S17009; K447*	Oita, Japan	AB553423	LC312243			LC312281
	TFM:S17003; K466*	Kanagawa, Japan	AB553424	LC312244	LC312355	LC312322	LC312282
	K467	Kanagawa, Japan	AB553425		LC312356		LC312283
	TFM:S17004; N52*	Kanagawa, Japan	AB553429	LC312245	LC312357	LC312323	LC312284
	TFM:S17002; K70*	Kanagawa, Japan	AB553408				LC312285
	S36	Kanagawa, Japan	LC312204				LC312286
	TFM:S17001; K230*		AB553416	LC312246			LC312287
	TFM:S17006; S71*	Kochi, Japan	LC312205		LC312358	LC312324	LC312288
***Tuber* sp. 6**	TFM:S17018; K152*	Ehime, Japan	AB553388	LC312226	LC312341	LC312308	AB553537
	K181	Yamanashi, Japan	AB553389	LC312227	LC312342	LC312309	LC312261
	K220	Hokkaido, Japan	AB553390	LC312228	LC312343		LC312262
	K222	Hokkaido, Japan	AB553391	LC312229	LC312344		LC312263
	TFM:S17019; K307*	Oita, Japan	LC312198	LC312230	LC312345	LC312310	LC312264
	K397	Kanagawa, Japan	AB553392	LC312231			LC312265
	TFM:S17020; K448*	Oita, Japan	AB553393		LC312346	LC312311	LC312266
	TFM:S17011; K464*	Miyagi, Japan	AB553394	LC312232		LC312312	LC312267
	TFM:S17012; K465*	Miyagi, Japan	AB553395	LC312233	LC312347	LC312313	LC312268
	N45	Oita, Japan	AB553396				LC312269
	N82	Hokkaido, Japan	AB553397	LC312234	LC312348		LC312270
	TFM:S17017; S72*	Kochi, Japan	LC312199		LC312349	LC312314	LC312271
	TFM:S17014; S4*	Kyoto, Japan	LC312200	LC312235	LC312350	LC312315	LC312272
	TFM:S17015; S17*	Hyogo, Japan	LC312201	LC312236		LC312316	LC312273
	TFM:S17016; S23*	Okayama, Japan	LC312202	LC312237	LC312351	LC312317	LC312274
	TFM:S17013; S27*	Chiba, Japan	LC312203	LC312238	LC312352		LC312275
***T*. *himalayense***	YR1-4	Yongren, Yunnan, China	LC312206	LC312247	LC312359	LC312326	LC312296
	YR1-6	Yongren, Yunnan, China	LC312207	LC312248	LC312360	LC312327	LC312297
	YM1-1	Yimen, Yunnan, China	LC312208	LC312249	LC312361	LC312330	LC312292
	YM1-2	Yimen, Yunnan, China	LC312209	LC312250			LC312293
	MY5-1	Miyi, Sichuan, China	LC312210	LC312251	LC312362		LC312295
	MY5-2	Miyi, Sichuan, China	LC312211	LC312252	LC312363		LC312294
	HP1-3	Huaping, Yunnan, China	LC312212	LC312253		LC312328	LC312298
	HP1-6	Huaping, Yunnan, China	LC312213	LC312254		LC312329	LC312299
	SHD1-2	Shidian, Yunnan, China	LC312214			LC312331	LC312291
	SHD2-2-14	Shidian, Yunnan, China	LC312215		LC312364	LC312332	LC312290
***T*. *indicum***	BSH1-11	Baoshan, Yunnan, China	LC312216	LC312255	LC312365	LC312337	LC312302
	BSH1-12	Baoshan, Yunnan, China	LC312217	LC312256	LC312366	LC312338	LC312303
	HD6-15	Huidong, Sichuan, China	LC312218	LC312257	LC312367	LC312335	LC312300
	HD6-16	Huidong, Sichuan, China	LC312219	LC312258	LC312368	LC312336	LC312301
	SM1-1	Songming, Yunnan, China	LC312220		LC312369	LC312339	LC312304
	SM1-2	Songming, Yunnan, China	LC312221			LC312340	LC312305
	ysh1-7	Yongsheng, Yunnan, China	LC312222		LC312370	LC312333	LC312306
	ysh1-8	Yongsheng, Yunnan, China	LC312223			LC312334	LC312307
***T*. *formosanum***	KUN-HKAS62628 (holotype)	Ho-she, Nantou, Taiwan	JN655530				
	KUN-HKAS62629	Ho-she, Nantou, Taiwan	LC312224	LC312259		LC312325	
	KUN-HKAS48268 (paratype)	Ho-she, Nantou, Taiwan	GU979048				
	KUN-HKAS79547.1	Ho-she, Nantou, Taiwan	LC312225	LC312260			LC312289
	KUN-HKAS79547.2	Ho-she, Nantou, Taiwan					

For Japanese specimens, asterisks indicate the samples used for morphological observations.

Details about the specimens of *T*. *indicum* and *T*. *himalayense* were shown in Feng et al. [15].

### Morphological observations

For Japanese specimens, we recorded ascomata size, external ornamentation shape, and colors following the Munsell System using mostly fresh specimens. Microscopic features of fresh and dried specimens were observed from slide preparations in 5% KOH. Photographs were taken under a light microscope; then, size of the fully matured ascospores and asci, and peridium thickness were measured using PhotoRuler 1.1 (http://hyogo.inocybe.info/_userdata/ruler/help-eng.html). For scanning electron microscopy (SEM), spores were scraped from the gleba and put directly onto an SEM stub with double-sided tape, coated with gold-palladium, and photographed with a HITACHI S-4800 (Hitachi Ltd., Tokyo, Japan).

Morphological analyses were conducted on 43 specimens, of which 20, 18, and 5 originated from Japan, China, and Taiwan, respectively (Table 1). To compare ascospore morphology of Japanese specimens with those of their allied taxa (*T*. *indicum*, *T*. *himalayense*, and *T*. *formosanum*), we arbitrarily selected 10 to 15 asci from specimens of each species and counted the numbers of spores on asci under light microscopes. Then, we measured ascospore length, width, length/width ratio (Q), and spine height from light microscope images; and breadths of spine bases were measured from SEM images. All measurements were recorded using PhotoRuler 1.1. Finally, spine height and spine bases were statistically compared among species based on Tukey–Kramer honestly significant difference test with R statistical software (http://www.r-project.org) after conducting a one-way ANOVA.

### DNA extraction, PCR amplification, and sequencing

Total DNA was extracted from approximately 1 mg glebal tissue of each fresh or dried ascomata using a DNeasy Plant Mini Kit (Qiagen, Valencia, California). The ITS region was amplified by PCR using the universal primers ITS1F [29] and ITS4 [30]. We also amplified two phylogenetically informative genes for the genus *Tuber* using two primer pairs, Bt2a/Bt2b [9,31] for beta-tublin (β-tublin) and EF1αTuber_f/EF1αTuber_r [32] for translation elongation factor 1-α (TEF1-α). For MAT loci, we targeted gene markers that encode a protein with an alpha domain (MAT1-1-1) in MAT1-1 and a protein with a DNA-binding domain of high mobility group (MAT1-2-1) in MAT1-2. The primer pairs i3 or i11/i12 were used for MAT1-1-1, and i5/i13 was used for MAT1-2-1 [20]. For the PCR amplification, we used the TaKaRa Ex Taq kit (Takara, Otsu, Japan), following the manufacture’s recommendations. PCR conditions were an initial denaturation as 95°C for 3 min, followed by 30 cycles of 95°C for 30 sec, 55°C for 30 sec, and 72°C for 2 min, with final extension at 72°C for 10 min. PCR products were purified with ExoSAP-IT (Affymetrix, Santa Clara, CA, USA) according to the manufacturer’s instructions. The purified PCR products were bi-directionally sequenced using the same primers that were used for PCR amplification. Sequencing was performed using an ABI3130xl automated sequencer (Applied Biosystems, Foster City, California) with a BigDye Terminator 3.1 Cycle Sequencing Kit (Applied Biosystems, Foster, CA, USA) following the manufacturer’s instructions.

### Phylogenetic analyses

Phylogenetic analyses were conducted based on single-locus (ITS, TEF1-α, β-tublin, MAT1-1-1, or MAT1-2-1) and concatenated multi-locus (ITS, TEF1-α, and β-tublin) datasets with an outgroup taxon, *T*. *melanosporum*. We aligned each dataset using MAFFT 7 [33] with default settings. Poorly aligned sites were identified using Gblocks 0.91B [34]. In this analysis, the minimum block-length was set to five, gaps were allowed in conserved blocks, and all other parameters were set to default. All identified ambiguous sites were excluded before phylogenetic analyses.

For MAT1-1-1 and MAT1-2-1 datasets, maximum likelihood (ML) analyses were conducted with PhyML 3.0 [35] under the TN93 and TN93+I models, respectively, which were selected by Smart Model Selection (SMS) implemented in PhyML. SH-like appropriate likelihood ratio test (SH-aLRT) was used to evaluate branching support. The ML trees were displayed by MEGA 7 [36]. We further conducted Bayesian phylogenetic analyses with MrBayes 3.2.6 [37]. In the Bayesian analyses, we applied the HKY model as the alternative model for each dataset (HKY85 for MAT1-1-1 and HKY+I for MAT1-2-1) because the best fit model of sequence evolution (TN93) can not be implemented in MrBayes 3.2.6. Two independent runs of four chains were conducted for 1,000,000 metropolis-coupled Markov chain Monte Carlo (MCMC) generations by sampling every 100th tree until the standard deviations of the split frequency became < 0.01. The log files of MrBayes were analyzed using Tracer 1.6 [38] to check the effective sample sizes (> 100). The first 10% of the sampled trees were discarded as burn-in. The remaining trees for each dataset were used to construct a 50% majority rule consensus tree, and the consensus trees were visualized with FigTree 1.4 [39]. We conducted the same phylogenetic analyses for ITS, TEF1-α, and β-tubulin. The consensus trees were visualized with MEGA 7. The complete alignment file was deposited in TreeBASE (Accession No. 21333).

To conduct a multi-locus phylogenetic analysis, the congruence among the three loci (ITS, TEF1-α, and β-tubulin) was checked by comparing the topology between individual phylogenetic trees based on the three loci [40,41]. Because there were no conflicting nodes among phylograms with higher branch support (>70% in aLRT), we combined ITS, β-tubulin, and TEF1-α datasets to make a superalignment for ML and Bayesian phylogenetic analyses. For ML analysis, the GTR+G+I model was used. For Bayesian analysis, a separate substitution model was applied for each locus (HKY+G+I for ITS and TEF1-α; HKY for β-tubulin). ML and Bayesian analyses were conducted using the above-mentioned software and settings.

### Nomenclature

The electronic version of this article in Portable Document Format (PDF) in a work with an ISSN or ISBN will represent a published work according to the International Code of Nomenclature for algae, fungi, and plants, and hence the new names contained in the electronic publication of a PLOS ONE article are effectively published under that Code from the electronic edition alone, so there is no longer any need to provide printed copies.

In addition, new names contained in this work have been submitted to MycoBank from where they will be made available to the Global Names Index. The unique MycoBank number can be resolved and the associated information viewed through any standard web browser by appending the MycoBank number contained in this publication to the prefix http://www.mycobank.org/MB/. The online version of this work is archived and available from the following digital repositories: PubMed Central and LOCKSS.

## Results

### Morphological analysis

**Fig 1:**
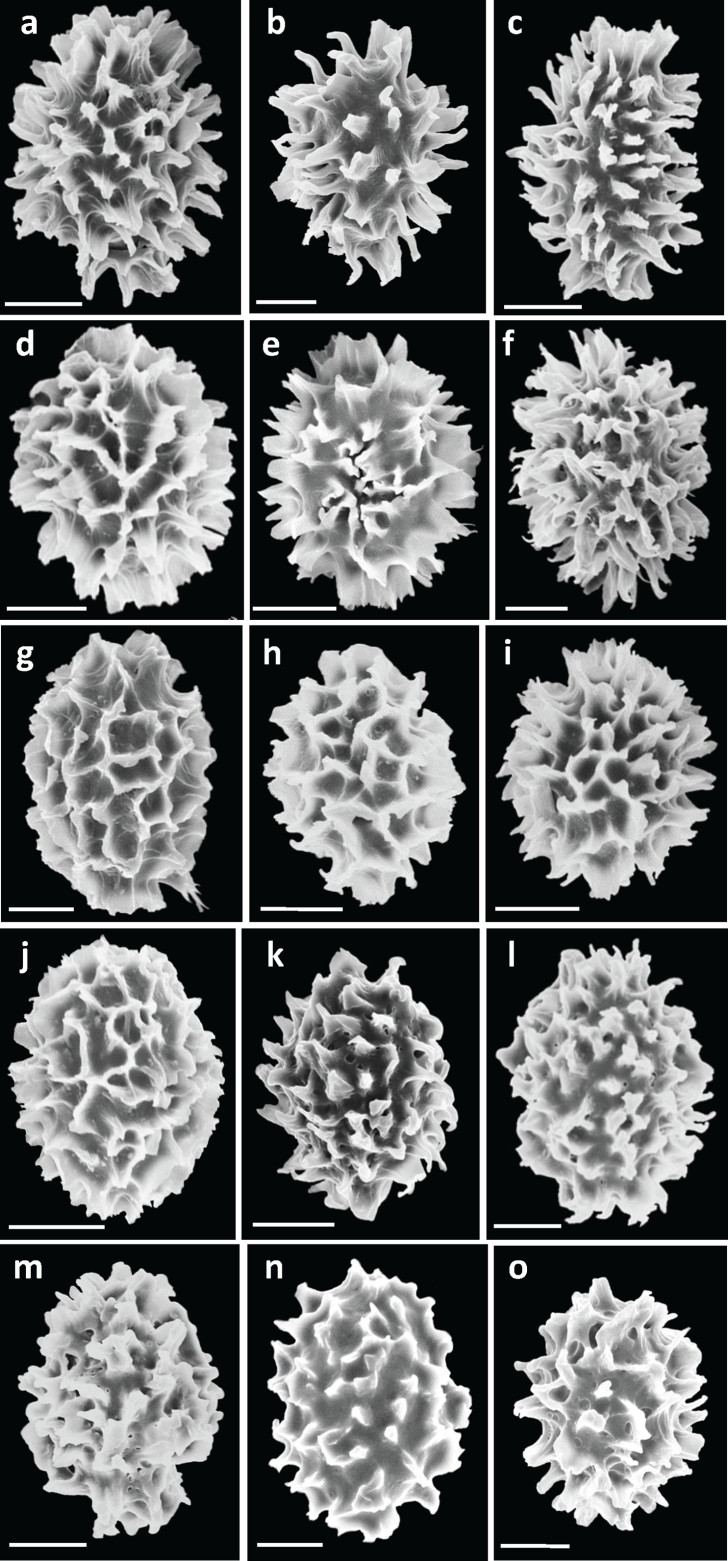
SEM images of ascospores for Asian black truffles, showing the details of ornamentation. A–C: *Tuber longispinosum* (A: K70, B: K209, C: K466), D–F: *Tuber* sp. 6 (D: K152, E: S4, F: S23), G–I: *T*. *formosanum* (G: HKAS48268 paratype, H: HKAS62628 holotype, I: HKAS79547), J–L: *T*. *himalayense* (J: HP1-3, K: MY5-1, L: SHD1-1-1), M–O: *T*. *indicum* (M: YSH1-8, N: YR1-4, O: HD6-16). Bars = 10 μm.

**Fig 2:**
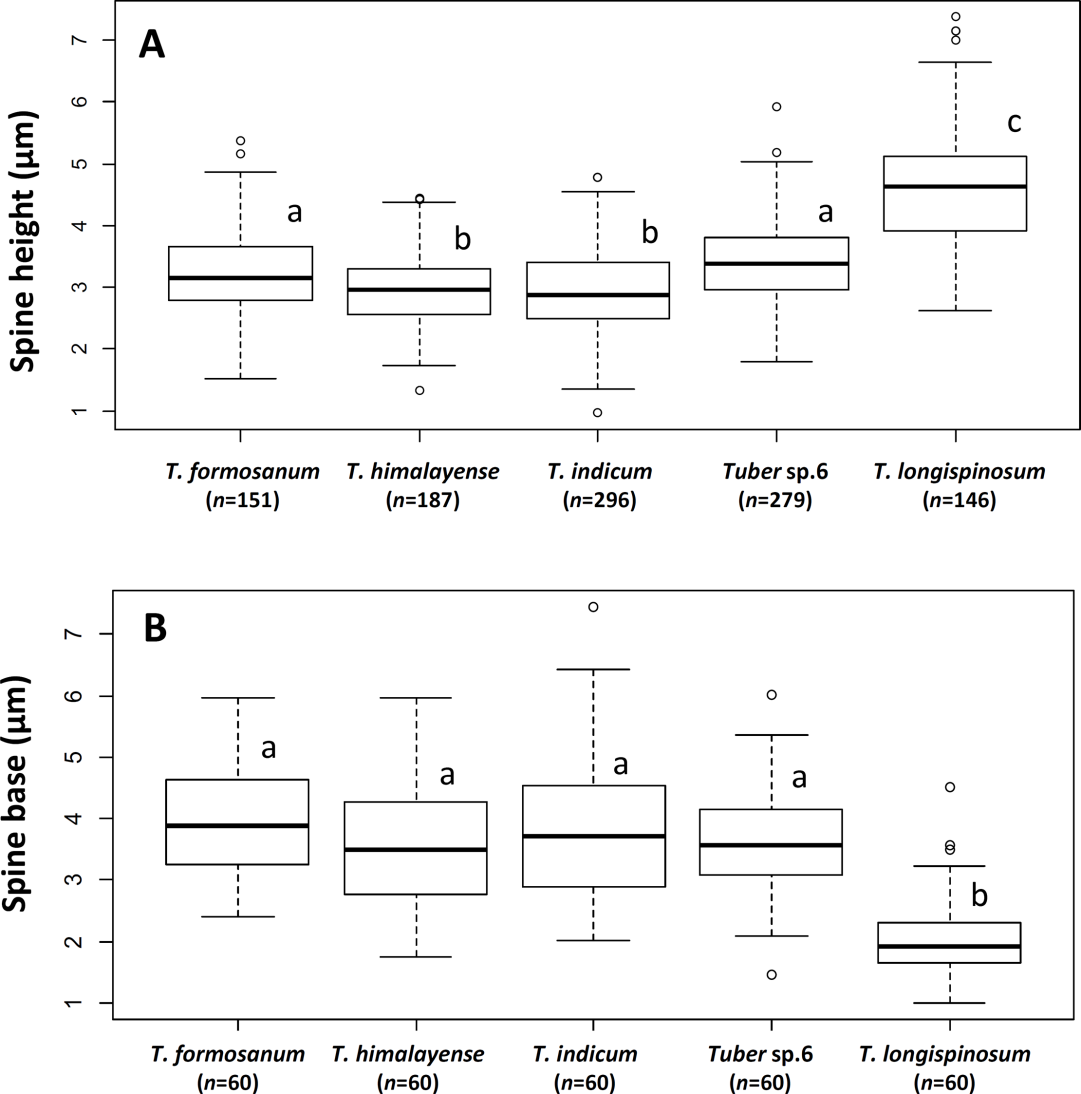
**Ascospore spine height of four-spored asci (A) and width of spine bases (B), for the studied taxa.** Different letters above boxes indicate significant differences between mean according to Tukey–Kramer honestly significant difference test (*P* < 0.01).

**Fig 3:**
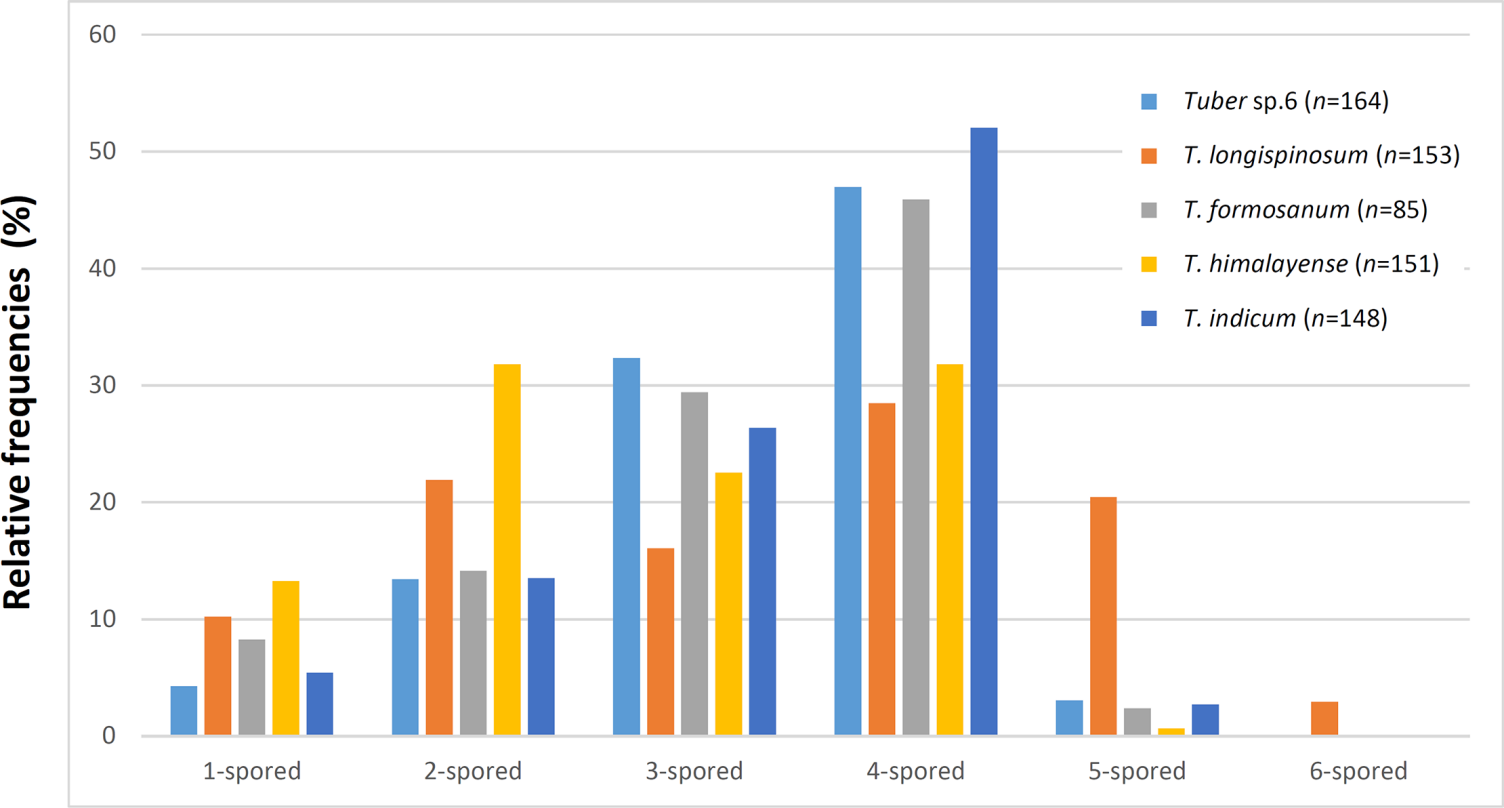
Relative frequencies of asci with one to six ascospores per ascus for the studied taxa.

Spore ornamentations were classified into three different types: spiny, partial reticulate (have both reticulum and spine on single ascospore), and spiny-reticulate (Fig 1). *Tuber* sp. 6 had all three types, of which the spiny spore was most abundant. Spine bases are wide and prone to fusion, forming a pseudoreticulum. *Tuber* sp. 7 had only spiny ascospores, the spines of which were sharp with narrower bases (2.1 ± 0.7 μm) than *T*. *indicum* (3.9 ± 1.3 μm), *T*. *himalayense* (3.6 ± 1.1 μm), *T*. *formosanum* (3.9 ± 0.9 μm), and *Tuber* sp. 6 (3.6 ± 0.9 μm) (mean ± SD, Fig 2, S1 Table). Although the spiny-reticulate ascospore was dominant in *T*. *formosanum*, spiny and partial reticulate ornamentation types were also observed. Ascospores of *T*. *himalayense* and *T*. *indicum* were mainly classified as spiny or partial reticulate ornamentation types, but the spiny-reticulate ornamentation type was observed in some *T*. *himalayense* ascospores. The number of spores per ascus ranged from one to six, and four-spored asci were most abundant, with 28–52% relative frequency for each species (Fig 3; S1 Table). Five-spored asci were rare in *T*. *formosanum*, *T*. *himalayense*, *T*. *indicum*, and *Tuber* sp. 6 (0.7–3.0%), but were rather frequently found in *Tuber* sp. 7 (20%). Spore length and width, and spine height generally became smaller with increasing numbers of spores per ascus, but there was no relationship between Q values and numbers of spores per ascus (S1 Table). Spore length and width, and Q values mostly overlapped among the putative species, regardless of spore numbers per ascus (S1 Table).

### Phylogenetic analyses

**Fig 4:**
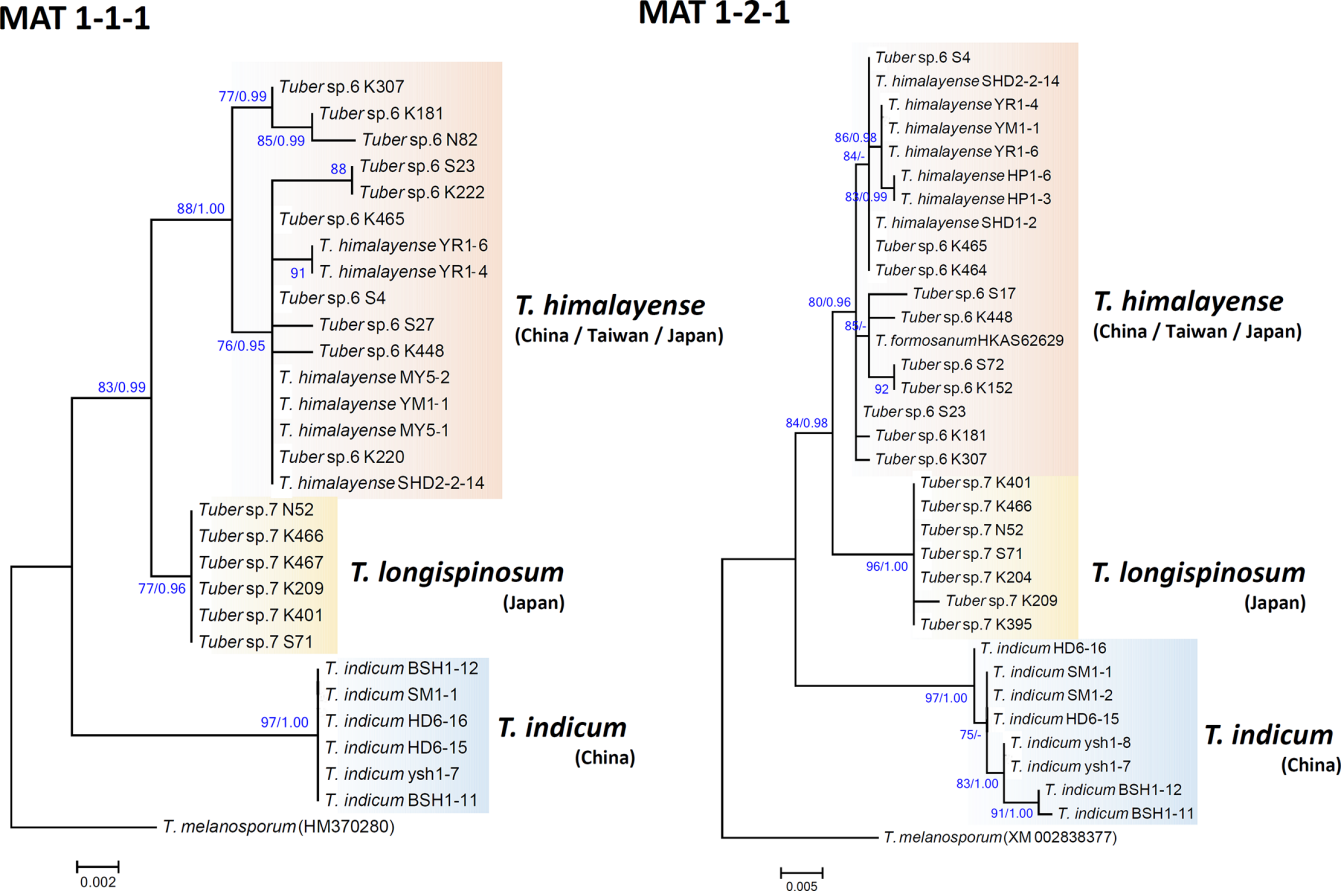
Phylogenetic relationships among Asian black truffles based on MAT1-1-1 and MAT1-2-1 sequences. The phylogram was obtained by maximum likelihood inference under the TN93 model. SH-aLRT values and Bayesian posterior probabilities are shown as ML/BPP.

We successfully amplified the MAT1-1-1 locus for 10 of 16 *Tuber* sp. 6 specimens, 6 of 13 *Tuber* sp. 7 specimens, 6 of 8 *T*. *indicum* specimens, and 6 of 10 *T*. *himalayense* specimens. Unfortunately, no MAT1-1-1 sequence was obtained from *T*. *formosanum* specimens. For the MAT1-2-1 locus, 10 of 16 *Tuber* sp. 6 specimens, 7 of 13 *Tuber* sp. 7 specimens, 7 of 10 *T*. *himalayense* specimens, 8 of 10 *T*. *indicum* specimens, and 1 of 5 *T*. *formosanum* specimens were successfully amplified. The sequence matrix of the MAT1-1-1 locus contained 30 sequences and 564 aligned bases, of which 49 bp were identified as poorly aligned by Gblocks 0.91b and thus were excluded from further analyses. The resultant MAT1-1-1 alignment was 515 bp. The MAT1-2-1 matrix contained 34 sequences and 741 bp aligned bases, of which 91 bp were identified as poorly aligned by Gblocks. After removing the poorly aligned sites, the resultant MAT 1-2-1 alignment was 650 bp. For both MAT1-1-1 and MAT1-2-1 loci, ML and Bayesian analyses yielded similar tree topologies; thus, only an ML tree is shown in Fig 4. Three distinct clades were recognized in both MAT1-1-1 and MAT1-2-1 phylograms, regardless of inference type (ML or Bayesian). In MAT1-1-1 phylogram, all *Tuber* sp. 6 sequences clustered with *T*. *himalayense*, and formed a sister relationship with a clade of *Tuber* sp. 7 sequences with high branch support (83/0.99). In MAT1-2-1, *Tuber* sp. 6 formed a clade with *T*. *formosanum* and *T*. *himalayense*, and formed a sister taxon to *Tuber* sp. 7 (84/0.98). *T*. *indicum* sequences formed a monophyletic clade that was positioned basally within the *T*. *indicum* complex in both phylograms.

**Fig 5:**
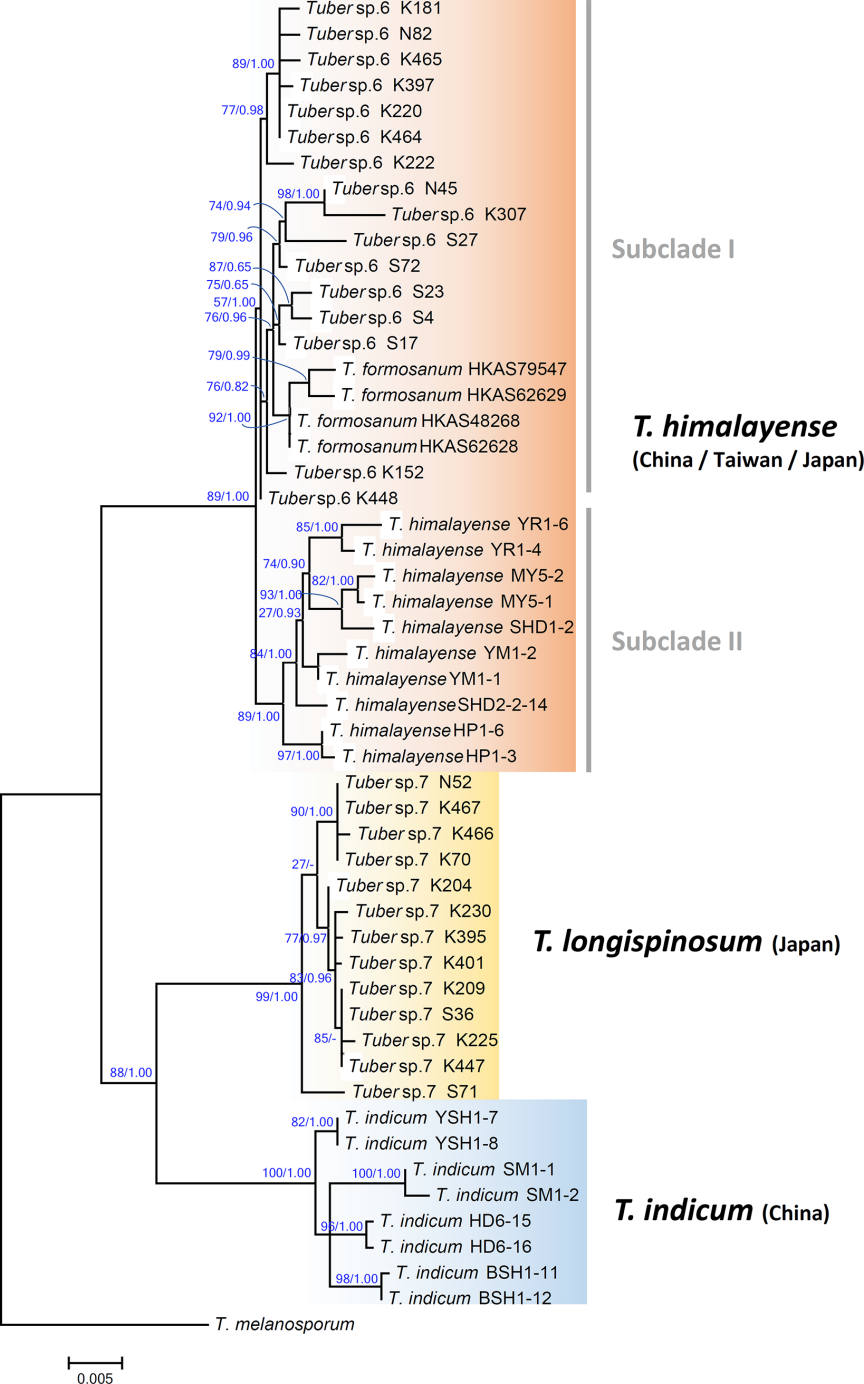
Phylogenetic relationships among Asian black truffles based on three combined datasets (ITS, β-tublin, and TEF1-α). The phylogram was obtained by maximum likelihood inference under the TN93+I model. SH-aLRT values and Bayesian posterior probabilities are shown as ML/BPP.

Because we found phylogenetic incongruence between two MAT loci and three concatenated DNA loci (ITS, TEF1-α, and β-tubulin), phylogenetic trees were reconstructed based on two MAT loci and the three combined loci, separately. The concatenated aligned matrix was 1,708 bp, including 541 bp of ITS, 409 bp of β-tubulin, and 758 bp of TEF1-α sequences, after removing poorly aligned sites with Gblocks. The three-locus phylogeny resolved three major lineages in the *T*. *indicum* species complex, with high branch support in both ML and Bayesian analyses: (1) *T*. *indicum*, (2) *Tuber* sp. 7, and (3) *T*. *himalayense* with *T*. *formosanum* and *Tuber* sp. 6 (Fig 5). These three lineages were also resolved in single-locus ITS, β-tublin, or TEF1-α phylograms (S1, S2 and S3 Figs). The *T*. *himalayense* clade consisted of two subclades, one of which was composed of *Tuber* sp. 6 and *T*. *formosanum* sequences, and the other was composed of *T*. *himalayense*. The *Tuber* sp. 7 sequences formed a monophyletic lineage in both ML and Bayesian analyses with high branch support (99/1.00), and formed a sister relationship with *T*. *indicum* sequences.

### Taxonomy

**Fig 6:**
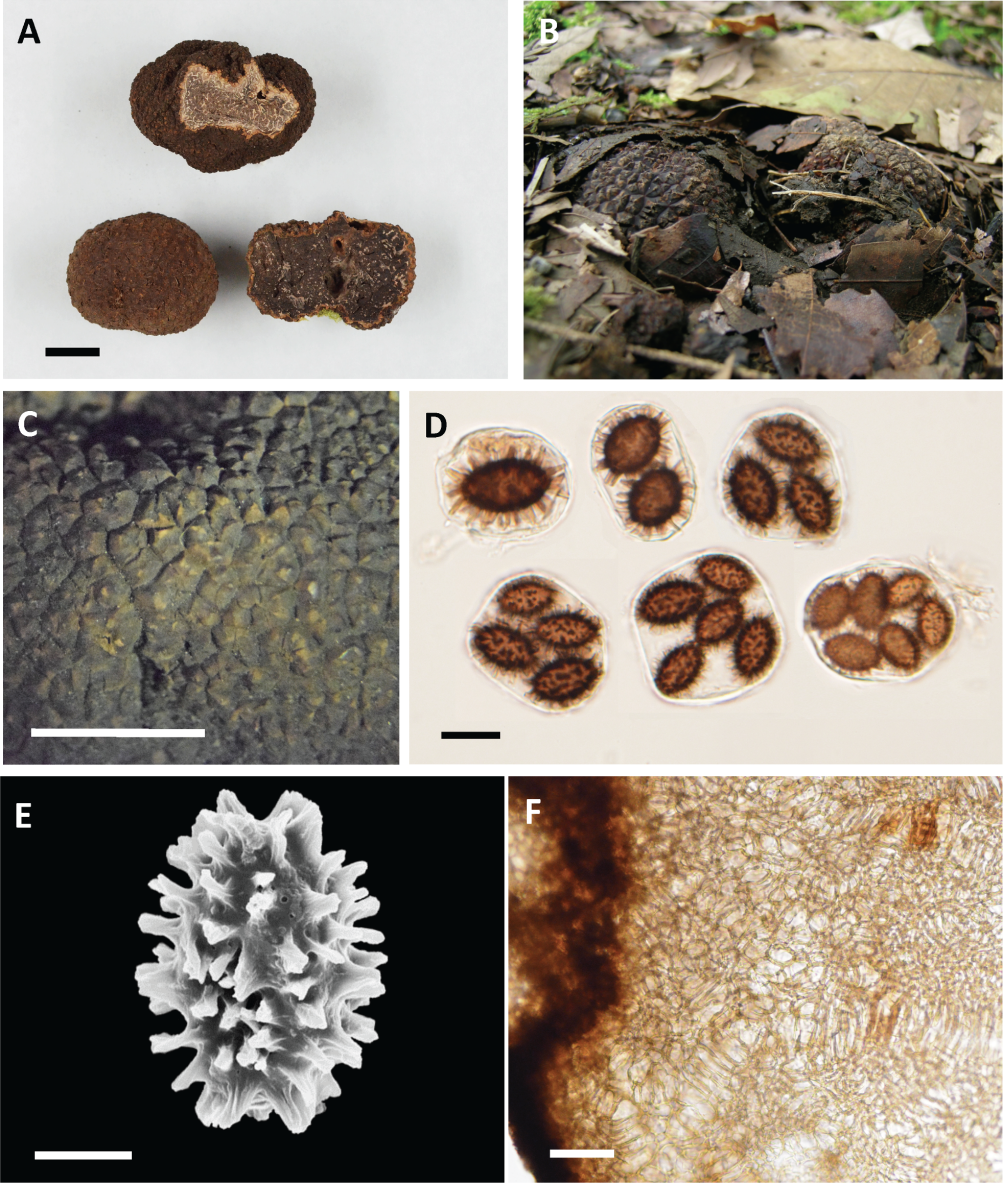
*Tuber longispinosum* photographs (holotype, TFM: S17009). A. Dried ascomata (bar = 1 cm). B. Fruit bodies photographed in the field. C. Peridial warts, (bar = 3 mm). D. Asci and ascospores (bar = 30 μm). E. Ascospore (bar = 10 μm). F. Peridium in cross section (bar = 50 μm).

***Tuber longispinosum***A. Kinosh. sp. nov. (Fig 6A–6F). [urn:lsid:mycobank.org:names:MB 821786].

Diagnosis: Differing from *T*. *indicum* and *T*. *himalayense* in ascospore ornamentation consisting of spines that are unconnected, and narrow at the base; and its significantly long spines.

Holotype: JAPAN, OITA Prefecture, under *Quercus acutissima* Carruth., 7 Oct 2006, collected by Hiromi Sasaki, K447 (TFM: S17009).

Ascomata subglobose, brown to dark greyish, 20–70 mm in diam. Peridium warty, two layers, the outer layer pseudoparenchymatous and composed of irregular cells. Gleba whitish when young, becoming grayish brown to blackish at maturity with yellowish cream to white veins. Asci 1–5(–6)-spored, 59–72 × 50–59 μm, subglobose to broadly ellipsoid. Ascospores ellipsoid to subglobose with spiny ornamentation, brown to dark brown, (15–)21–35(–41) × (12–)15–26(–30) μm in diam excluding ornamentation.

Etymology: *longispinosum* (Lat.), referring to “with long spine” from its spore ornamentation (Japanese name “*Iboseiyoshoro*” from *ibo* = warts, *seiyoshoro* = Japanese name for the genus *Tuber*).

Ascomata: hypogeous, 20–70 mm in diam., subglobose and slightly lobed, firm, brown (10R 4/8) to dark greyish (7.5YR 8/18), with low polygonal warts, 3–7 ridges, up to 900 μm high. Odor: aromatic, similar to seaweed or laver boiled in soy sauce when mature. Peridium: 400–800 μm thick, variable, pseudoparenchymatous, composed of two layers: outer layer 100–200 μm thick, composed of irregular or ellipsoidal cells, 7–28 × 5–18 μm, with thick dark brown walls of 1–2 μm; inner layer 200–600 μm thick, composed of hyaline to yellowish, polygonal cells 5–15 × 5–10 μm that merge with glebal tissue of interwoven hyphae. Gleba: solid, whitish when young, becoming greyish brown to blackish at maturity, marbled with distinct, yellowish cream to whitish, meandering veins that merge at many points. Glebal tissue of interwoven hyphae: 3–8 μm broad with scattered cells, gelatinized, inflated up to 10 μm. Asci: typically subglobose to broadly ellipsoid, occasionally ellipsoid, variable depending on the number of spores, 59–72 × 50–59 μm (*n* = 177), rarely stipitate, 1–5(–6)-spored. Ascospore: ellipsoid, whitish or hyaline (glass-like) when young, becoming light brown (5Y 8/3) to dark brown (5YR 9/4) at maturity, 31–41 × 22–30 μm, Q = 1.3–1.6 (1-spored, *n* = 18); 21–38 × 16–29 μm, Q = 1.0–1.8 (2-spored, *n* = 78); 19–34 × 15–26 μm, Q = 1.1–1.7 (3-spored, *n* = 70); 15–33 × 13–22 μm, Q = 1.0–2.1 (4-spored, *n* = 146); 16–31 × 12–20 μm, Q = 1.1–1.8 (5-spored, *n* = 118); 15–26 × 13–18 μm, Q = 1.0–1.8 (6-spored, *n* = 24), excluding ornamentation, typically free spines, 3–7 (–12) μm (*n* = 430) in height with 1–4-μm bases.

Habitat and distribution: The fruiting period is from July to January. Woodland or forest edges under *Abies* (Pinaceae), *Carpinus* (Betulaceae), and *Castanopsis* and *Quercus* (Fagaceae).

Additional specimens examined (paratypes): JAPAN, KANAGAWA Prefecture, Atsugi City, under *Carpinus tschonoskii* Maxim. and *Quercus serrata* Murray, 9 Dec 2007, collected by Hiromi Sasaki, K230 (TFM: S17001); Hayama-cho, under *Castanopsis sieboldii* (Makino) Hatsushima ex Yamazaki et Masiba and *Quercus glauca* Thunb., 30 Dec 2002, collected by Hiromi Sasaki, K70 (TFM: S17002); Ibid., 25 Oct 2004, collected by Kazuhide Nara, N52 (TFM: S17004); Ibid., 23 Dec 2005, collected by Hiromi Sasaki, K466 (TFM: S17003); SHIZUOKA Prefecture, Izu City, under *Abies firma* Sieb. Et Zucc., *Quercus salicina* Blume, and *Q*. *serrata*, 31 Jan 2003, collected by Hiromi Sasaki, K395 (TFM: S17005); KOCHI Prefecture, Umaji Village, under *Q*. *glauca* and *Q*. *serrata*, 3 Feb 2017, S71 (TFM: S17006); EHIME Prefecture, Matsuyama City, 14 Oct 2006, collected by Fumitaka Nagao, K209 (TFM: S17007); OITA Prefecture, Saiki City, under *Quercus* spp., 17 Oct 2003, collected by Yoichi Sunada, K401 (TFM: S17008); Yufuin-cho, 29 Sep 2007, collected by Atsuko Hadano and Hiromi Sasaki, K225 (TFM: S17010).

Additional comments: *T*. *longispinosum* have more five-spored asci than the other species, but the frequency of five-spored asci varies depending on specimens; observations of two or more specimens are needed. Sakae Takayama and Shoichi Yoshimi first found a black truffle in Japan and identified it as *T*. *indicum* (Japanese name “*Iboseiyoshoro*”) [42]. The ascospores exhibit surface ornamentation with conspicuously long spines that were 4–8 (–10) μm high with 4 μm bases, which correspond to the *T*. *longispinosum* characters. Therefore, we assigned the Japanese “*Iboseiyoushoro*” to *T*. *longispinosum*.

***Tuber himalayense***B.C. Zhang & Minter, Trans. Br. Mycol. Soc. 91(4): 595 (1988).

**Fig 7:**
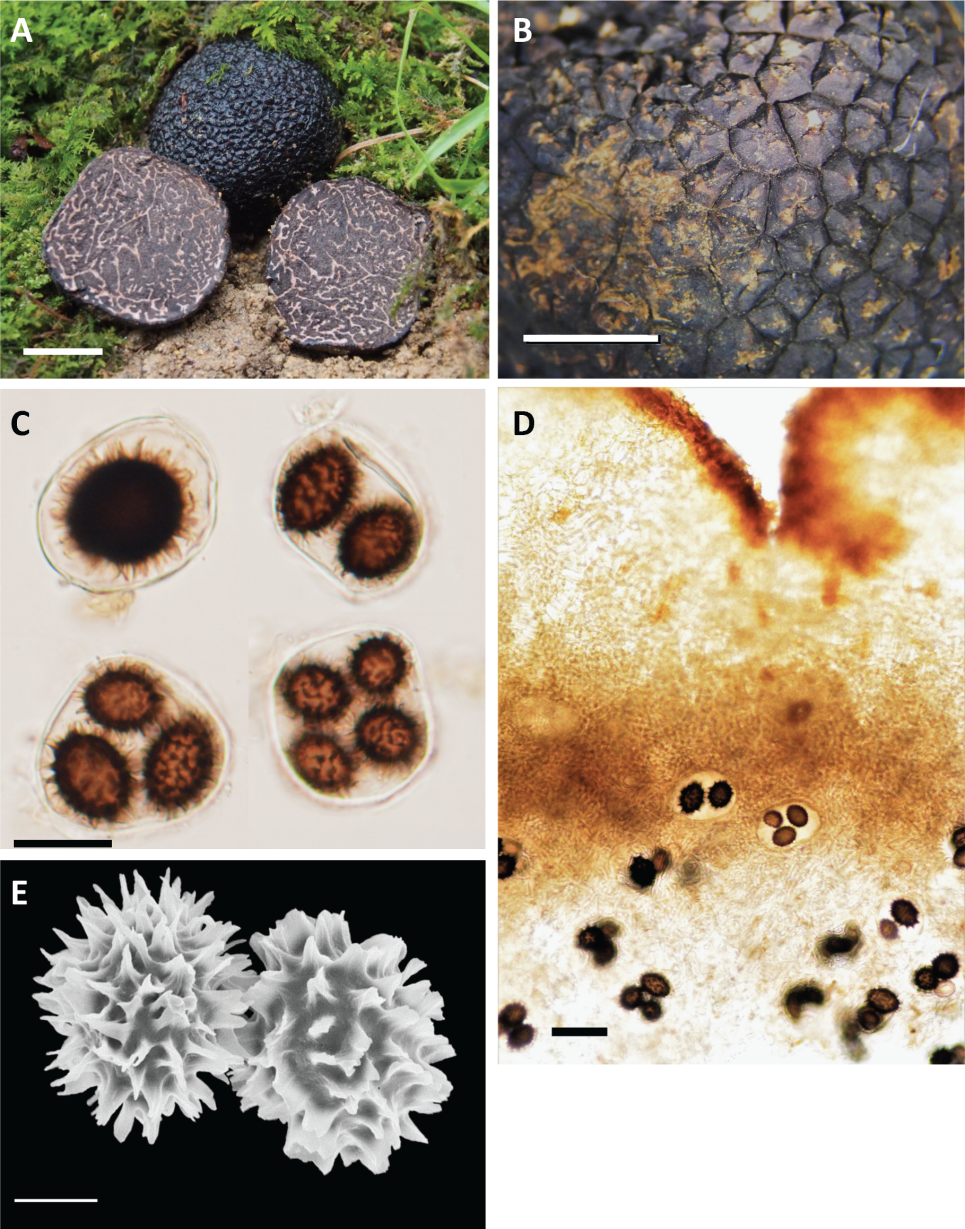
*Tuber himalayense* photographs (TFM: S17015). A. Ascomata (bar = 1 cm). B. Peridial warts, (bar = 3 mm). C. Asci and ascospores (bar = 30 μm). D. Peridium and glebal tissue in cross section (bar = 50 μm). E. Ascospore (bar = 10 μm).

MycoBank MB134661. Fig 7A–7E = *Tuber formosanum* H.T. Hu & Y. Wang, Mycotaxon 123: 296 (2013).

MycoBank MB563693

Etymology: Japanese name “*Asiakuroseiyoshoro*” from *Asia* = locality, *Kuro* = black, *seiyoshoro* = Japanese name for the genus *Tuber*).

Ascomata: hypogeous, 20–60 mm in diam, subglobose and slightly lobed, firm, brown (10R 4/8) to dark greyish (7.5YR 8/18), with low polygonal warts, 4–6 ridges, up to 500-μm high. Odor: aromatic, similar to seaweed or laver boiled in soy sauce when mature. Peridium: 400–800 μm thick, variable, pseudoparenchymatous, composed of two layers: outer layer 150–200 μm thick, composed of irregular or ellipsoidal cells 10–20 × 5–15 μm, with thick, dark brown 1–2-μm walls; inner layer 200–600-μm thick, composed of hyaline to yellowish, polygonal cells 5–15 × 5–10 μm that merge with glebal tissue of interwoven hyphae. Gleba: solid, whitish when young, becoming dark brown to blackish at maturity, marbled with distinct, whitish, meandering veins that merge at many points. Interwoven hyphae of glebal tissue: 3–7 μm broad with scattered cells, gelatinized, inflated up to 10 μm. Asci: typically subglobose to broadly ellipsoid, occasionally ellipsoid, variable depending on number of spores, 48–81 × 38–73 μm (*n* = 164), rarely stipitate, 1–4(–5)-spored. Ascospore: mostly ellipsoid, rarely globose, whitish or hyaline when young, becoming light brown (5Y 8/3) to dark brown (5YR9/4) at maturity, 31–41 × 24–35 μm, Q = 1.1–1.6 (1-spored, *n* = 7); 24–44 × 17–32 μm, Q = 1.0–1.6 (2-spored, *n* = 22); 17–37 × 15–33 μm, Q = 1.0–1.8 (3-spored, *n* = 154); 14–34 × 12–26 μm, 1.0–2.0 (4-spored, *n* = 279); 18–31 × 14–22 μm, Q = 1.0–1.9 (5-spored, *n* = 24), excluding ornamentation. Ornamentation with very variable: spines with free, partial reticulate, spiny-reticulate, and alveolate. Spines up to 2–7 (9) μm (*n* = 508) in height with 3–7-μm bases.

Habitat and distribution: North-western Provinces of India to southern China, Taiwan and Japan. In Japan, the fruiting period is from July to January. Woodland under *Betula* and *Carpinus* (Betulaceae); *Castanea*, *Castanopsis* and *Quercus* (Fagaceae); and *Abies* and *Pinus* (Pinaceae).

Specimens examined: JAPAN: MIYAGI Prefecture, Sendai City, Dec 2003, collected by Yoko Ando, K464 (TFM: S17011); Sendai City, 23 Oct 2005, collected by Yoko Ando, K465 (TFM: S170012); CHIBA Prefecture, Narashino City, under *Q*. *acutissima*, 15 Nov 2015, collected by Hiromi Kinoshita, S27 (TFM: S17013); KYOTO Prefecture, Kyoto City, under *Q*. *glauca* and *Q*. *serrata*, 6 Dec 2004, collected by Takashi Yamanaka and Keisuke Obase, S4 (TFM: S17014); HYOGO Prefecture, Sanda City, under *Q*. *glauca*, 27 Nov 2015, collected by Mitsuo Nabe and Michiyo Nabe, S17 (TFM: S17015); OKAYAMA Prefecture, Niimi City, under *Carpinus tschonoskii* and *Q*. *serrata*, 19 Dec 2015, collected by Hideo Hara, S23 (TFM: S17016); KOCHI Prefecture, Umaji Village, under *Q*. *glauca* and *Q*. *serrata*, 3 Feb 2017, S66 (TFM: S17017); EHIME Prefecture: Futami-cho, under *Castanopsis sieboldii* and *Quercus* sp., 24 Nov 2006 collected by Fumitaka Nagao, K152 (TFM: S17018); OITA Prefecture, Yufu City, 24 Oct 2008, collected by Atsuko Hadano and Eiji Hadano, K307 (TFM: S17019); Yufu City, under *Q*. *acutissima* and *Q*. *serrata*, 8 Oct 2006, collected by Hiromi Sasaki, K448 (TFM: S17020).

Additional comments: Hu [4] described *T*. *formosanum* from Taiwan as a distinct species based on morphological observation; subsequently, Qiao et al. [6] typified *T*. *formosanum* based on a newly collected sample, because there was no typification in the original description by Hu [4]. They denoted that *T*. *formosanum* differs from *T*. *indicum* by its asci with a short stipitate, spiny-reticulate ascospores and association with *Cyclobalanopsis glauca* (= *Quercus glauca*) [6]. However, we showed that *T*. *formosanum* is phylogenetically and morphologically indistinguishable from *T*. *himalayense* (= *T*. *indicum* group B) and *Tuber* sp. 6. Because *T*. *himalayense* was described by Zhang & Minter [7] before *T*. *formosanum* was described by Hu [4], we synonymize *T*. *formosanum* with *T*. *himalayense* (hereafter we call *Tuber* sp. 6 and *T*. *formosanum* as “*T*. *himalayense*”).

## Discussion

Phylogenetic analyses of the *T*. *indicum* complex have been conducted based on ITS, LSU, Protein Kinase C, β-tublin, mcm7, and TEF-1α sequences [8,9,13,14,15,43], and all analyses showed two distinct lineages referred to as *T*. *indicum* groups A and B. Here, we provide the first MAT phylogenies for the *T*. *indicum* complex, including Japanese specimens. Three independent lineages were resolved: *T*. *indicum*, *T*. *longispinosum*, and *T*. *himalayense*; this was also confirmed in the three-locus phylogeny (ITS, β-tublin, and TEF1-α). The *T*. *himalayense* clade was composed of specimens that had mainly spine or pseudoreticulum spore ornamentations, and some specimens exhibited a rather complete reticulum, such as the *T*. *himalayense* type specimen [K(M)33236] [7] (S1 Fig). Alternatively, the specimens that belonged to the *T*. *indicum* clade generally had the same morphological characters as those of the *T*. *himalayense* clade, but had no complete reticulum ornamentation. This corresponds to the characters of the *T*. *indicum* type specimen [7,14]. Thus, our phylogenetic and morphological analyses revealed that the specimens that belonged to *T*. *indicum* and *T*. *himalayense* clades were generally consistent with the findings of the previous studies.

### Taxonomy of Japanese black truffles in the *T*. *indicum* complex

*Tuber longispinosum* differed from the *T*. *indicum* and *T*. *himalayense* specimens based on three morphological traits. First, the specimens that belonged to the *T*. *indicum* and *T*. *himalayense* clade displayed multiple ornamentation types, whereas the specimens that belonged to the *T*. *longispinosum* clade were exclusively composed of spiny ascospores (Figs 1 and 6). Previous studies also reported that the specimens that belonged to the *T*. *indicum* and *T*. *himalayense* clades generally displayed high variation in spore ornamentation among or within specimens [8,19] (S2 Table). Second, the width of spine bases and spine height on *T*. *longispinosum* ascospores were significantly narrower and higher than those of the other species (Fig 2; S1 Table). This is also largely related to the spore ornamentation differences among the above-mentioned species [6,8,19]. Finally, Merenyi et al. [44] showed that the distribution of asci with different numbers of spores is a key character for distinguishing between *T*. *brumale* and *T*. *cryptobrumale*. We also provide evidence that the higher frequency of five-spored asci compared with other species is an important feature when distinguishing *T*. *longispinosum* from allied taxa (Fig 3). However, because the distribution of spores in different asci varies by specimen, two of the above-mentioned morphological characters need to be simultaneously checked when identifying by morphology alone.

Here, we revisited the phylogenetic relationships of Japanese *T*. *himalayense* (formerly *Tuber* sp. 6), which fell into a clade that included specimens from China and Taiwan (Figs 4 and 5). We found no clear morphological boundary among geographical origins of the specimens of *T*. *himalayense*. For example, although ascospores of Japanese specimens had mostly spiny ornamentations, their spines have broad bases, are sometimes fused with the adjacent spines, and forming a reticulum (Figs 1 and 7). These characters have also been confirmed not only in Chinese and Taiwaniese specimens but also in *T*. *indicum* [4,6,8,14,19]. We did not use the *T*. *himalayense* type specimen in this study because it was reported to be in poor condition [8,11,14]. However, a sequence (AY773356) from a specimen which is morphologically identical to the *T*. *himalayense* type specimen [14], clustered with the Japanese and Taiwaniese sequences in the ITS phylogeny (S1 Fig). Alternatively, the *T*. *himalayense* clade was divided into two subclades in the three-locus phylogeny (Chinese and Taiwanese-Japanese subclades in Fig 5). Therefore, we cannot completely exclude the possibility that the two subclades are independent species. However, until the presentation of more compelling evidence to the contrary, we consider Taiwaniese and Japanese specimens to represent *T*. *himalayense*.

### MAT genes are useful markers for elucidating *T*. *indicum* complex taxonomy

We successfully amplified and sequenced two MAT loci for Japanese specimens using the same primer sets as those that were developed for the *T*. *indicum* complex, which indicates that *T*. *longispinosum* and *T*. *himalayense* are also heterothallic. Moreover, three independent lineages were revealed by phylogenetic analyses, but the relationships among them are unclear because we found incongruent results between reproductive and non-reproductive genes (Figs 4 and 5). Although MAT genes are functional markers that are primary determinants of sexual compatibility, it is unclear to what extent the divergence level among strains affect the species recognition. Rather, mating compatibility and mutual recognition between strains of opposite mating types are mediated by the pheromone-receptor system [45]. These genes have already been identified in the *T*. *melanosporum* genome [20,21]. Therefore, to better understand the phylogenetic relationships and species distinction among Asian black truffles, more taxon sampling outside the *T*. *indicum* complex is needed, and analysis of the pheromone receptor gene could be explored for its utility.

### Biogeography of Asian black truffles

*Tuber longispinosum* and *T*. *himalayense* (samples of *Tuber* sp. 6) are probably associated with *Betula*, *Castanea*, *Carpinus*, *Quercus*, and *Pinus*; some of those trees are thought to have migrated from continental Asia to the Japanese Archipelago when the sea level was reduced and land bridges appeared during the Pleistocene (e.g., *Q*. *glauca* [46] and *Q*. *acutissima* [47]). Therefore, it is possible that the two truffle species migrated with their hosts from continental Asia, as was the case with *T*. *japonicum* [48]. A similar biogeographical scenario to that of *Boletus reticuloceps* [49] can also be inferred for *T*. *formosanum* in Taiwan Island; this species was considered an independent taxon because of its sole host plant [*Cyclobalanopsis glauca* (Thunb.) Oerst.] and distribution (Taiwan) [6,8,43]. However, Taiwan was connected to continental Asia between 1 and 0.015 Ma [50,51], and *C*. *glauca* has been considered a synonym of *Q*. *glauca* (The Plant List: http://www.theplantlist.org/). *Q*. *glauca* has a wide geographical distribution, extending from the southern slope of Himalaya to Taiwan and Japan [52,53]. The refugium in Taiwan has been estimated in the central part of the Island [54], which corresponds to the habitat of *T*. *formosanum* [4,6]. Thus, we suggest that common ancestors of *T*. *himalayense* migrated with host plants into Japan and Taiwan from continental Asia.

## Conclusions

Our study is the first to demonstrate that MAT loci are useful for species delimitation in *T*. *indicum* complex and the results recover similar topologies as shown in previous multilocus phylogenetic analysis. We were able to describe a new species of *Tuber* (*T*. *longispinosum*), based on morphological and phylogenetic data obtained from one of the *T*. *indicum* phylotypes. We could not find any morphological differences between *T*. *indicum* and *T*. *himalayense* specimens, regardless of their phylogenetic distinctiveness, and treat the second phylotype (*Tuber* sp.6) and *T*. *formosanum* as synonym of *T*. *himalayense*.

## Supporting information

S1 Fig Phylogenetic relationships among Asian black truffles based on rDNA ITS sequences. The phylogram was obtained by maximum likelihood inference under the HKY+G+I model. SH-aLRT values and Bayesian posterior probabilities are shown as ML/BPP.
(TIF)

S2 Fig Phylogenetic relationships among Asian black truffles based on β-tublin sequences. The phylogram was obtained by maximum likelihood inference under the TN93+I model. SH-aLRT values and Bayesian posterior probabilities are shown as ML/BPP.
(TIF)

S3 Fig Phylogenetic relationships among Asian black truffles based on TEF1-α sequences. The phylogram was obtained by maximum likelihood inference under the TN93+G+I model. SH-aLRT values and Bayesian posterior probabilities are shown as ML/BPP.
(TIF)

S1 Table Spore length and width, Q value, spine height, and breadth of spine bases for each species. Q values indicates ratio of length/width. Values is the mean; minimum and maximum values are between parentheses. *n = 60 for each species.
(TIF)

S2 Table Morphological characters of ascospores for known species. (TIFF)
