# Supplementary figures and images for: Using mating-type loci to improve taxonomy of the Tuber indicum complex, and discovery of a new species, T. longispinosum
Source: PLoS One. 2018 Mar 28;13(3):e0193745. doi: 10.1371/journal.pone.0193745 (PMC5874008; doi:10.1371/journal.pone.0193745)

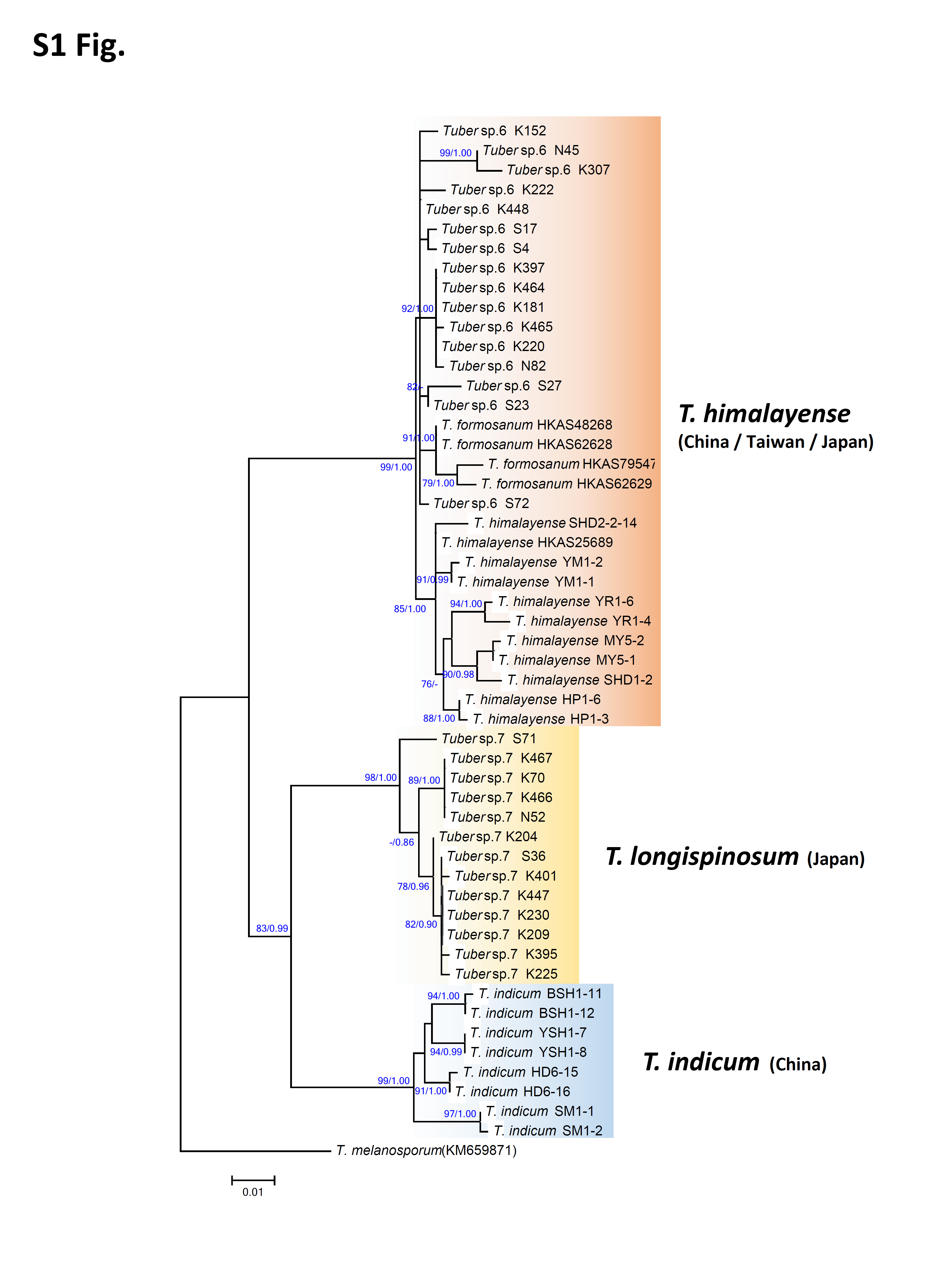

Supplement: S1 Fig — The phylogram was obtained by maximum likelihood inference under the HKY+G+I model. SH-aLRT values and Bayesian posterior probabilities are shown as ML/BPP. (TIF) [file pone.0193745.s001.tif]

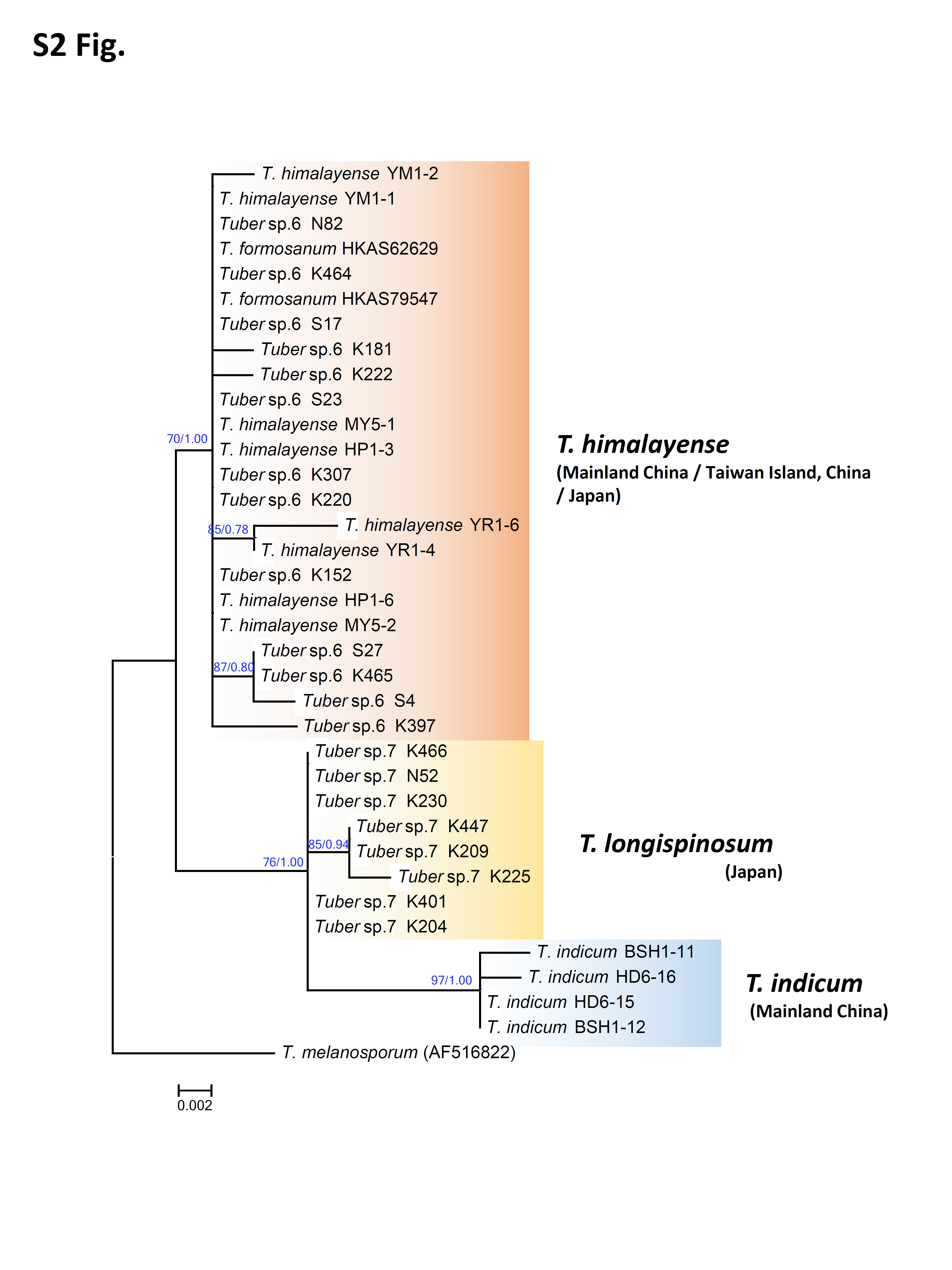

Supplement: S2 Fig — The phylogram was obtained by maximum likelihood inference under the TN93+I model. SH-aLRT values and Bayesian posterior probabilities are shown as ML/BPP. (TIF) [file pone.0193745.s002.tif]

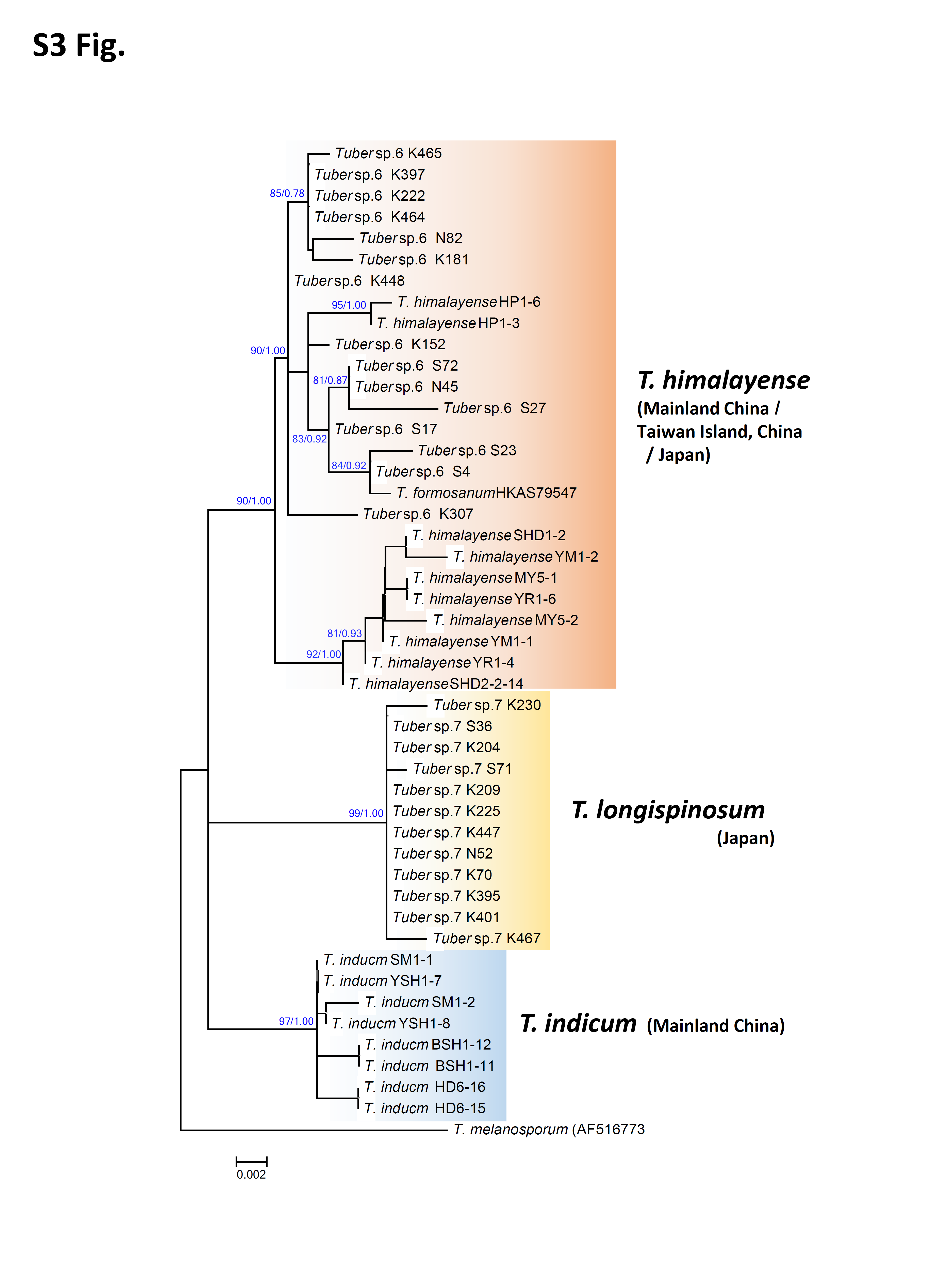

Supplement: S3 Fig — The phylogram was obtained by maximum likelihood inference under the TN93+G+I model. SH-aLRT values and Bayesian posterior probabilities are shown as ML/BPP. (TIF) [file pone.0193745.s003.tif]

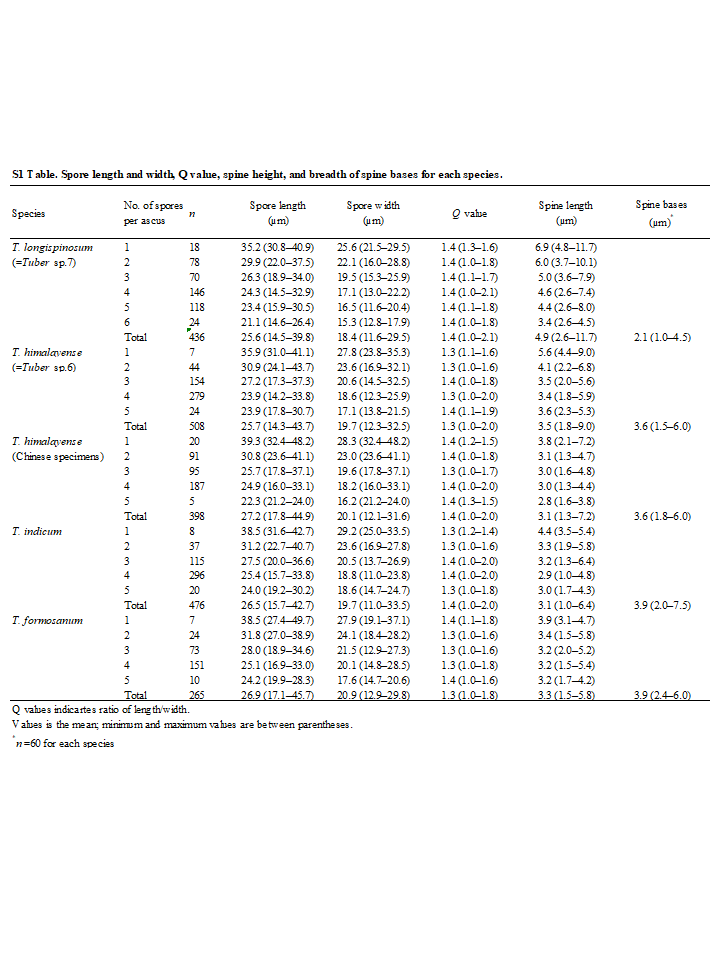

Supplement: S1 Table — Q values indicates ratio of length/width. Values is the mean; minimum and maximum values are between parentheses. *n = 60 for each species. (TIF) [file pone.0193745.s004.tif]
